# Anatomic Transtibial Tunnel Placement Using an Initial 6‐mm Drill Bit for Anterior Cruciate Ligament Reconstruction

**DOI:** 10.1002/atn2.70049

**Published:** 2026-06-17

**Authors:** Rafael Calvo Rodríguez, Fernando Martin Kommer, Robert Partarrieu Stegmeier, Consuelo Carrasco Vera, Miguel Carrasco Garrido, Rafael Calvo Mena, David Figueroa Poblete

**Affiliations:** ^1^ Department of Traumatology, Knee and Arthroscopy Unit Clinica Alemana Santiago Chile; ^2^ Faculty of Medicine Clinica Alemana Santiago ‐ Universidad del Desarrollo Santiago de Chile Región Metropolitana Chile

## Abstract

Precise anatomic tunnel placement is essential for restoring native knee biomechanics and minimizing graft failure in anterior cruciate ligament reconstruction. We describe a tibial tunnel drilling technique designed to enhance tunnel placement accuracy, reduce intra‐articular trauma, and preserve native tibial footprint remnants when indicated. Following standard arthroscopic visualization, tibial and femoral tunnel positioning is guided by established anatomic landmarks. Using a tibial guide, a guidewire is initially placed at the intended tibial site. If positioning is suboptimal, a 6‐mm tunnel is created, which provides a controlled working corridor for subsequent guidewire relocation within its margins. This step enables adjustment of tunnel trajectory with high precision. A definitive 10‐mm tunnel is then drilled at the corrected location. Notably, expansion from a 6‐ to 10‐mm tunnel using this technique only increases the overall cross‐sectional tunnel area within the tibia by ~5%, thereby maintaining bone stock and minimizing damage. This technique offers a reproducible method to refine tibial tunnel placement while mitigating the risks associated with repeated drilling and excessive tunnel enlargement.

## Graphical abstract

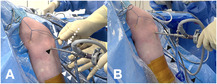

**VIDEO 1:** Presentation of technique for tibial tunnel drilling in anterior cruciate ligament (ACL) reconstruction. This video illustrates the key steps of the surgical technique. An initial 6‐mm drill bit is used to establish a preliminary tunnel, which serves as a guide. This allows for intraoperative assessment and fine‐tuning of the tunnel's position before upsizing to the final diameter. The technique is shown to reduce the “explosive” entry into the joint, enable precise anatomical placement, and minimize damage to the native ACL remnant. Video content can be viewed at https://doi.org/10.1002/atn2.70049.

Anterior cruciate ligament (ACL) reconstruction is among the most frequently performed procedures in orthopaedic surgery. In the USA, the annual incidence is approximately 70 reconstructions per 100,000 individuals.^1^ Revision ACL reconstruction remains technically demanding, with technical errors representing one of the most frequently reported causes of graft failure. Data from the Multicenter ACL Revision Study showed that 24% of revisions were attributable to technical errors, of which 37% involved tibial tunnel malposition.^2^ Anatomic positioning of tunnels is paramount to restore native knee biomechanics and reduce graft failure.^3^ We describe a technique for tibial tunnel drilling, which allows for more precise tibial tunnel placement, less traumatic entrance to the knee joint, and less damage to the ACL remnant in cases where preservation is desired.

## SURGICAL TECHNIQUE

**FIGURE 1:**
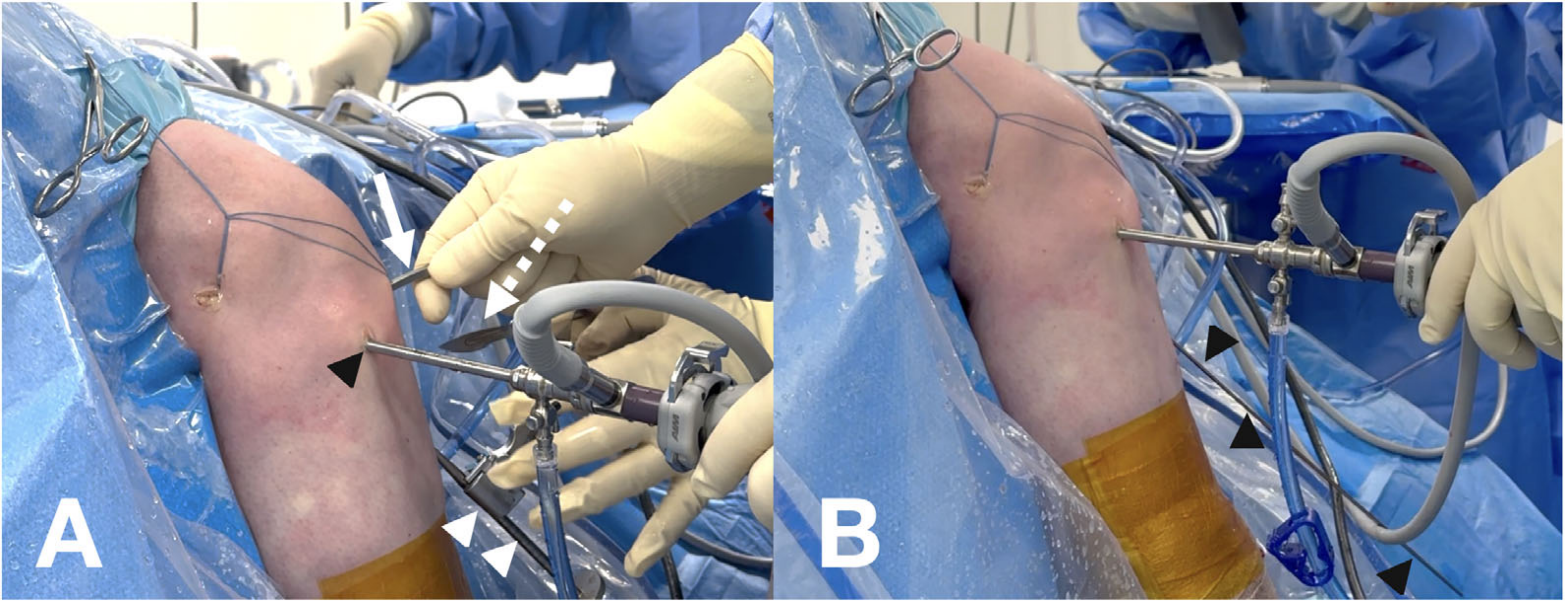
Patient positioned supine on an orthopaedic table that allows the knee to hang free, with a lateral post supporting the right knee and a pneumatic tourniquet applied to the thigh at 250 mmHg. The knee is flexed to 90°. In (A), white arrow indicates the tibial handle guide through anteromedial portal, black arrow indicates the 4.0‐mm arthroscope with 30° scope through anterolateral portal, dotted white arrow indicates a scalpel just before performing the skin incision to the entry of the tibial tunnel, and white arrowheads indicate the guide sleeve through which the 2.4‐mm guide pin is inserted. In (B), black arrowheads show the 2.4‐mm guide pin already inserted.

The patient is positioned supine on an orthopaedic table, with a lateral post supporting the knee and a pneumatic tourniquet applied on the thigh at 250 mmHg. The knee is flexed to 90° (Figure 1). The procedure begins with graft harvesting with a closed stripper (AR‐1278L, Arthrex, Naples, FL) of the semitendinosus and gracilis tendons through an anteromedial incision located 5 cm distal to the joint line. The graft is then prepared on a dedicated workstation using high‐strength sutures, yielding a quadrupled construct. In accordance with the findings of Magnussen et al., our group prefers a minimum graft diameter of 8 mm.^4^ If this cannot be achieved, the graft is prepared as a quintupled construct or augmented with an allograft (hybrid technique).

Standard anterolateral and anteromedial portals are then established, and a diagnostic arthroscopy is performed. Associated lesions (meniscal or chondral) are addressed at this stage. Next, the intercondylar notch is prepared using a shaver for adequate debridement. Rarely, a burr is used for notchplasty, particularly in chronic lesions, female patients, or in cases of a narrow notch. The torn ACL is debrided, taking care to preserve functional remnant fibers if possible. To conclude this initial stage, both anatomical footprints are debrided and clearly marked.

The anatomical footprints of the ACL are then identified. For the femoral side, we follow the description by Lubowitz et al. (43% of the proximal‐distal distance of the lateral intercondylar notch wall, with the tunnel radius positioned 2.5 mm anterior to the posterior articular margin).^5^ For the tibial side, the description by Verma et al. is used (2 ± 0.49 mm anterior to the posterior border of the anterior horn of the lateral meniscus).^6^

**FIGURE 2:**
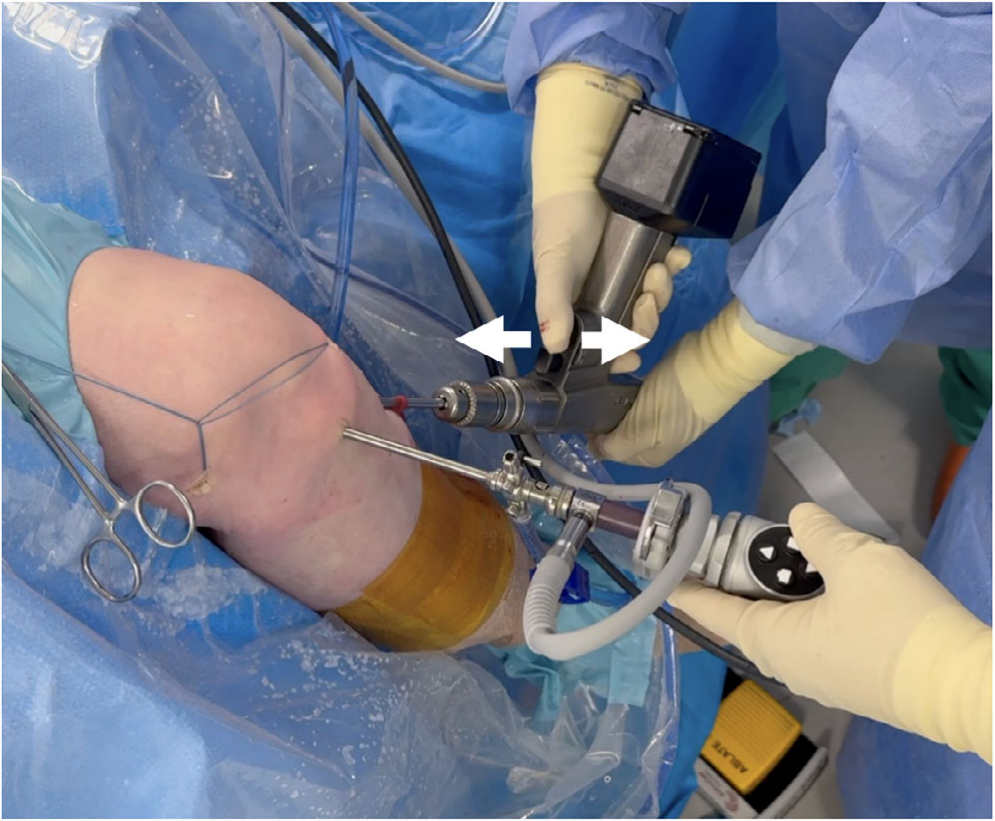
Operating room overview of the guide positioning and drilling during surgery of a right knee. Drilling can be performed in a pulsatile or intermittent manner to enhance control and minimize a traumatic or abrupt entry into the joint. Theoretically, this may also help reduce friction and heat generation. White arrows show how triggering of the power drill is used in an intermittent manner. The left hand of the surgeon covers the back of the power drill to avoid dropping the guide pin.

**FIGURE 3:**
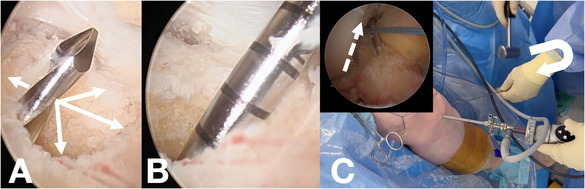
Repositioning the guide allows controlled relocation of the final tunnel within the 6‐mm tract, enabling millimetric corrections and adjustment of tibial tunnel placement. In image (A,B) the viewing portal is anteromedial of a left knee, whereas in (C) is the anterolateral portal of a right knee. In (A), the guide pin is medial, and in (B), the guide is moved more lateral and posterior within a 6‐mm tibial tunnel. White arrows in (A) depict how the 2.4‐mm guide can move around the 6‐mm tunnel to make the desired correction. The guide pin can be gently tapped with a hammer into the femur (as seen in (C), dotted white arrow shows the guide pin already tapped and big white arrow depicts hammering) or stabilized with a surgical clamp (e.g., Kelly clamp) through the anterolateral portal to maintain its position for correction for the final drilling.

**FIGURE 4:**
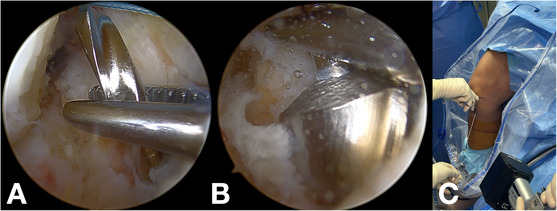
(A) The arthroscopic view of the guidewire, which can be adjusted to the desired position within the previously drilled 6‐mm tunnel and then held in place by a clamp, aiding in maintaining the planned trajectory for the subsequent 10‐mm drilling, as shown in (B). (C) The intraoperative view of the clamp introduced through the anteromedial arthroscopic portal of a right knee. In image (A,B) the viewing portal is anterolateral.

**FIGURE 5:**
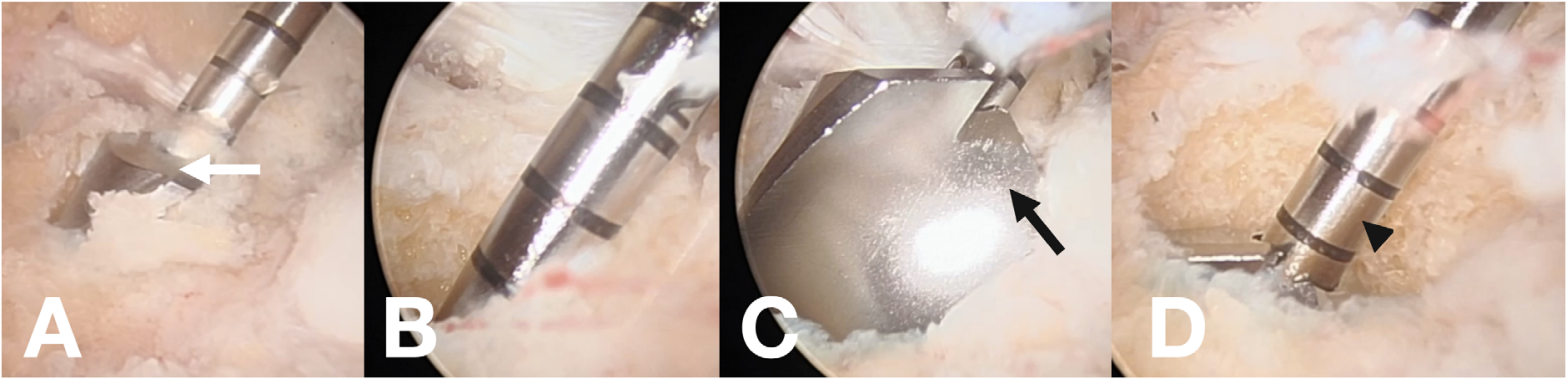
Less traumatic drilling sequence. Drilling can be performed in a pulsatile or intermittent manner to improve control and reduce the risk of abrupt joint entry. In (A), the 6‐mm drill bit is used (white arrow). In (B), the guide pin is moved lateral and posterior. (C) The image shows a 10‐mm drill bit (black arrow). Final tunnel is seen in (D) with the guide pin in place (black arrowhead). Images from a left knee. In images (A) through (D), the viewing portal is the anteromedial portal.

**FIGURE 6:**
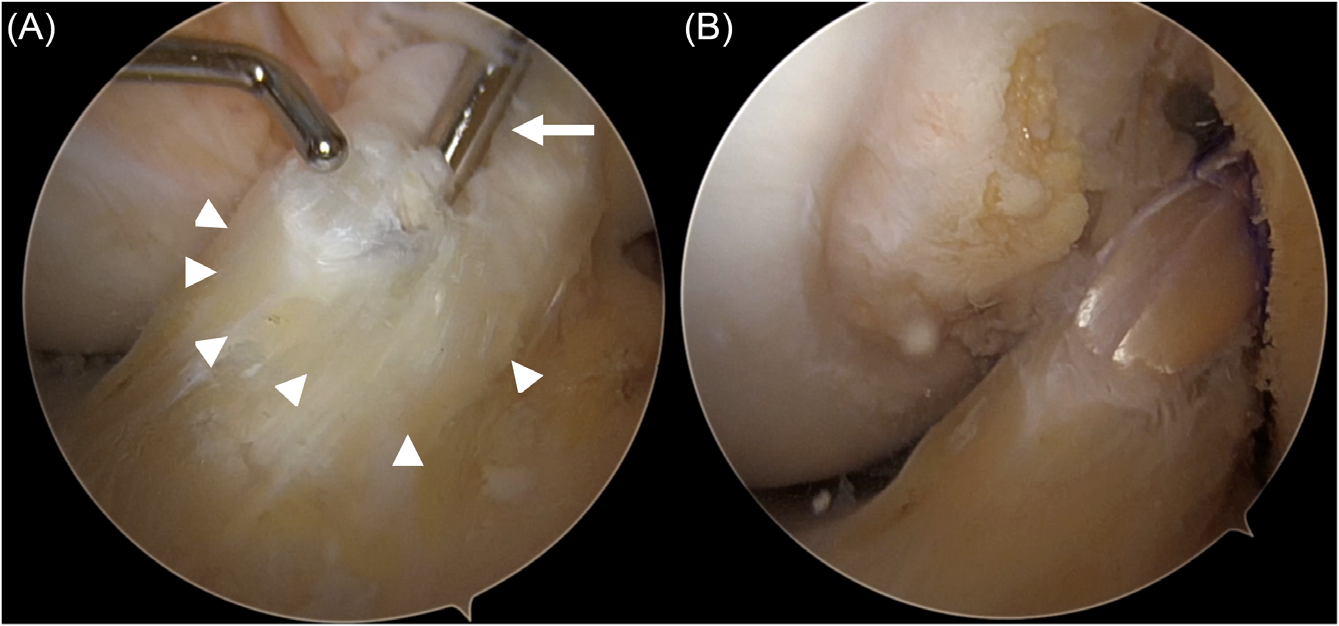
Remnant preservation is performed with an initial 6‐mm drill bit. In (A), white arrowheads show bulking under the remnant due to reaming, and the white arrow shows the 2.4‐mm guide pin already in place. In (B), the final hamstring graft with remnant preservation. Images from a left knee. The viewing portal is the anterolateral portal.

The tibial marking guide (Infinity Guide System, Conmed Corporation, USA, or ACL Hook AR‐1510T, Arthrex, Naples, FL) is introduced through the anteromedial portal targeting the previously described anatomical ACL tibial footprint. The guide is fixed at an angle between 55° and 60°. A standard guide sleeve is positioned through the incision made for graft harvesting (Infinity Guide System, Conmed Corporation, USA, or AR‐1204F‐24I 2.4‐mm guide pin sleeve, Arthrex, Naples, FL). A standard guide pin of 2.4 mm is then advanced through the sleeve, under arthroscopic visualization, ensuring its exit at the tibial footprint where the marking guide was placed. The guide handle and sleeve are removed, while leaving the pin in position. If the trajectory is deemed suboptimal, a 6‐mm tunnel is drilled over the pin with cannulated drill bits (AR1206‐11, Arthrex, Naples, FL) and compatible power drilling system (Stryker System 8, Stryker, USA) (Figure 2). This allows controlled relocation of the final tunnel within the 6‐mm tract, thereby enabling millimetric corrections. Two maneuvers can be employed to maintain the guidewire and preserve the desired new trajectory: gentle tapping of the wire into the bone or stabilization using a clamp (Figures 3 and 4). This allows us to modify tibial tunnel placement (Figure 3), make a less traumatic entrance to the knee joint (Figure 5), and better preserve native ACL remnant in cases where preservation is desired (Figure 6).

**FIGURE 7:**
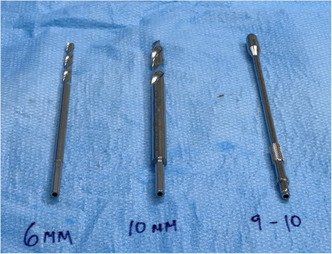
Cannulated drill bits of 6 and 10 mm and a blunt dilator, from left to right. This instrumentation is required to safely and accurately perform the technique.

**FIGURE 8:**
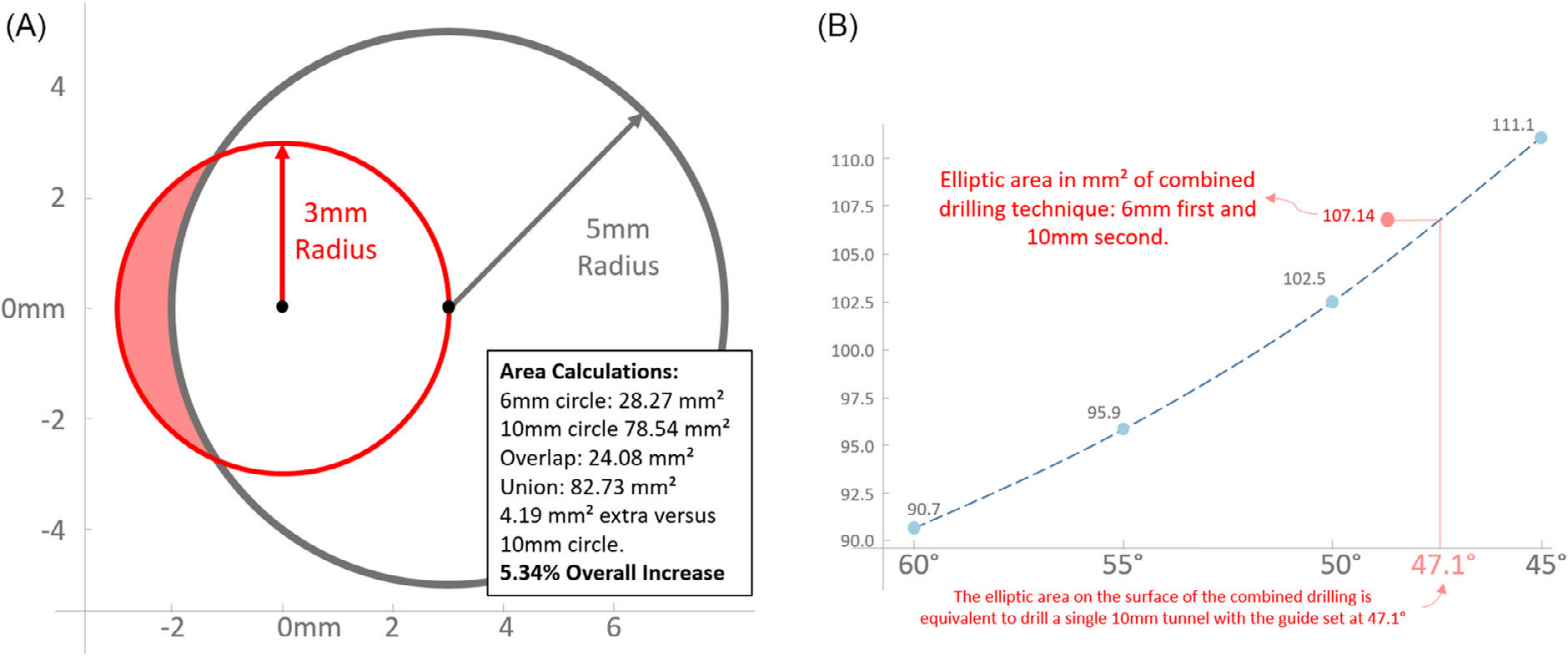
(A) Diagram illustrating the increase in cross‐sectional area (in red) when the final 10‐mm tunnel (gray circle) is drilled at the edge and parallel to the axis of the initial 6‐mm tunnel (red circle). (B) Graph showing the resulting elliptical surface area (vertical axis, mm^2^) produced by oblique drilling on the tibial surface with a 10‐mm diameter, plotted against the drilling angle set on the hand guide (horizontal axis; a higher angle indicates a more vertical tibial tunnel). The combined elliptical area generated by the sequential drilling technique (6 mm followed by 10 mm in a corrected position) starting with the guide set at 55° is 107.14 mm^2^, which is equivalent to drilling a single 10‐mm tunnel with the guide set at approximately 47°.

**FIGURE 9:**
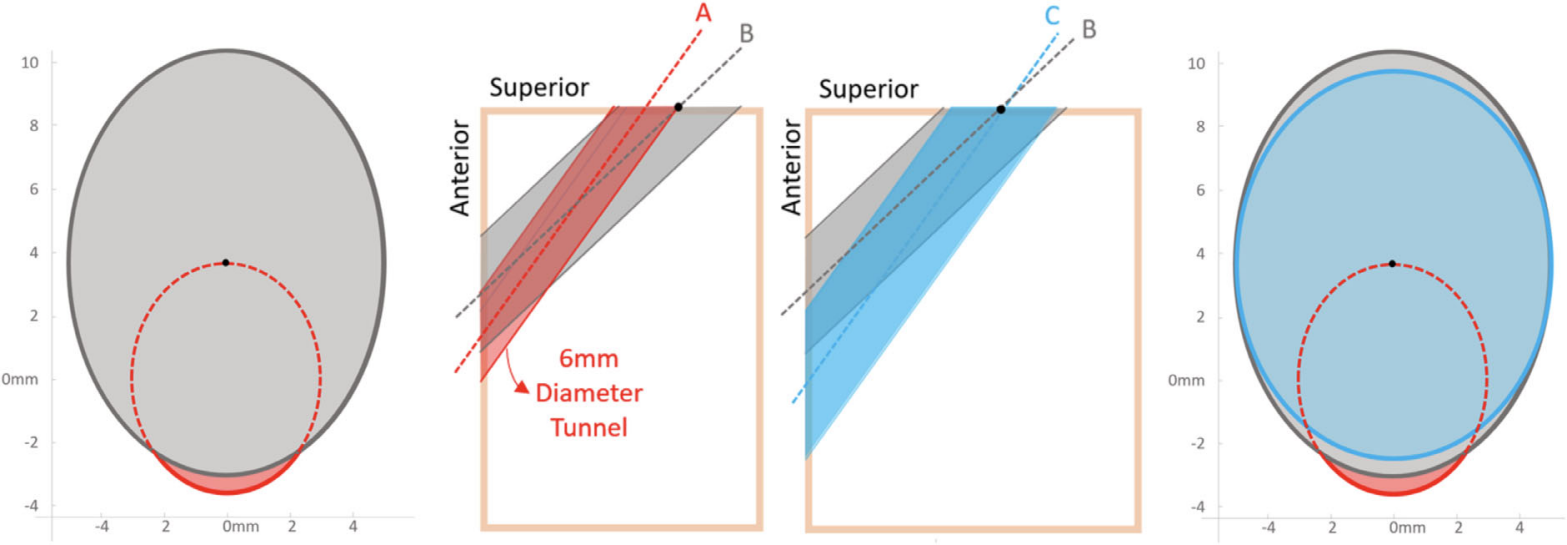
Simplified model illustrates the calculation of surface area increase when the final 10‐mm tunnel is drilled at the edge of the initial 6‐mm tunnel, considering the elliptical apertures formed at the superior tibial surface. On the left, a superior view shows the ellipse produced by the 6‐mm drilling with the guide set at 55° (in red) and the ellipse generated by a 10‐mm drill oriented from the upper edge of the entry point toward the most posterior trajectory (approximately 48.5°, in gray), which represents the configuration generating the largest potential mismatch. On the right, the overlapping areas are depicted: the 6‐mm tunnel at 55° (red), the 10‐mm tunnel at 48.5° or corrected path (gray), and the ellipse corresponding to a 10‐mm tunnel drilled directly at 55° (blue). At the center, a 2‐dimensional lateral schematic shows tunnel trajectories, where A (red and red dashed line) represents the 6‐mm tunnel with the guide at 55°, B (gray and gray dashed line) corresponds to the path creating the greatest mismatch, and C (blue and blue dashed line) represents a single 10‐mm tunnel drilled without correction. The calculated areas of the ellipses at the superior tibial surface are as follows: 6‐mm tunnel with guide at 55° (red) = 34.52 mm^2^; 10‐mm tunnel with guide at 48.5° (gray) = 104.87 mm^2^; overlap (red and gray) = 32.24 mm^2^; union (red + gray) = 107.14 mm^2^; and single 10‐mm tunnel with guide at 55° (blue) = 95.88 mm^2^. The extra area is therefore 11.26 mm^2^, corresponding to an overall increase of 11.74%. Nevertheless, since the guide is often set between 45° and 60° in clinical practice, the surface area increase at the superior tibial exit can be considered negligible.

**FIGURE 10:**
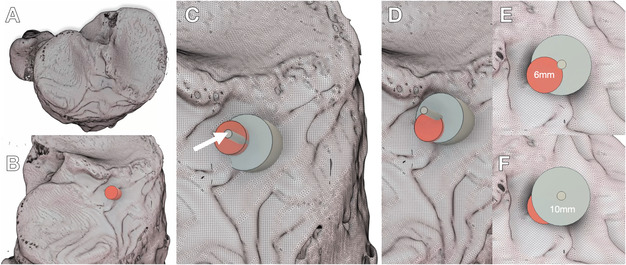
Three‐Dimensional model with a superior oblique view of the proximal tibia with the 6‐mm tunnel in red and 10 mm with 2.4‐mm guide wire in gray. (A) Superior view of a three‐dimensional model of the tibia. (B) A 6‐mm tunnel track in red. In (C), the white arrow points to the 2.4‐mm guide pin that can be moved around the 6‐mm red track or tunnel. (D) The amount of correction that can be made in comparison to the position of the gray cylinder in (C). (E) Axial view of the cylinder or track formed by the 6‐mm drill and the guide pin with the 10‐mm cylinder. (F) The 5% area increase in red versus a single 10‐mm tunnel in gray.

The definitive diameter is drilled, and the tunnel is subsequently dilated to match the graft diameter with a blunt dilator (AR‐1854‐8/11 blunt dilators, Arthrex, Naples, FL) (Figure 7). Moving the final tunnel within the 6 mm, one only increases the tibial tunnel area within the tibia by approximately 5% if a final 10‐mm tunnel is drilled in the worst‐case scenario (at the edge of the initial tunnel) (Figures 8 and 9). The cross‐sectional area of the tunnel is slightly increased, as displayed in the model shown in Figures 8 and 10. In contrast, the exit of the tunnel at the tibial surface forms an elliptical aperture, making its calculation more complex. Using a simplified geometric model for elliptical areas, we found that, on the superior surface, a 6‐mm tunnel drilled with the hand guide set at 55°, followed by a tangent 10‐mm tunnel starting from the uppermost point of the first ellipse on the lateral face and running obliquely within the initial tunnel's volume until exiting at the border of the first ellipse on the superior face, yields a combined aperture of 107.14 mm^2^. This represents an increase of 11.26 mm^2^ (11.74%) compared with a single 10‐mm tunnel drilled at 35° (95.88 mm^2^) (Figure 9). However, this combined area is equivalent to drilling a single 10‐mm tunnel with the guide set at approximately 47° (Figure 8).

Graft fixation on the tibial side is performed with an interference screw, applying continuous traction on the graft with the knee positioned at 20° of flexion and slight external tibial rotation. The complete technique can be seen in Video 1, and other relevant pearls and pitfalls are highlighted in Table 1.

**TABLE 1:** Pearls and Pitfalls of Tibial Tunnel Placement Using an Initial 6‐mm Drill Bit for Anterior Cruciate Ligament Reconstruction

**Pearls**	**Pitfalls**
While drilling, use your free hand to support the back of the power drill to prevent dropping the cannulated guide wire	The tibial guide pin could be dropped accidentally when drilling
Cannulated drill bits and compatible power drill are required	Make sure 6‐mm cannulated drill bit is available beforehand (we use a 2.4‐mm guide pin)
The guide pin can be gently tapped with a hammer into the femur or stabilized with a surgical clamp (e.g., Kelly) through the anterolateral portal to maintain its position for correction and subsequent final drilling	Avoid excessive levering inside the tunnel during drilling or the while the drill bit is inserted, as this may damage the tunnel walls
Drilling can be performed in a pulsatile or intermittent manner to enhance user control and avoid even more a traumatic or abrupt entry into the joint. Theoretically, this may also help to reduce friction and heat generation	

## DISCUSSION

This technique has 3 main advantages and some disadvantages that are summarized in Table 2. The advantages are that it reduces intra‐articular trauma by mitigating the “explosive” entry effect of the drill bit. Second, it enables precise correction of the tibial tunnel, thereby enhancing anatomic accuracy. Third, in cases in which ACL remnant preservation is desired, it minimizes disruption of the native tibial footprint. One disadvantage is the slight increase in tunnel area produced by sequential drilling (e.g., 6‐mm tunnel drill followed by a 10 mm one). Nonetheless, this enlargement is negligible, with calculations showing only a ~5% increase when expanding from 6 to 10 mm.

**TABLE 2:** Advantages and Disadvantages of Tibial Tunnel Placement Using an Initial 6‐mm Drill Bit for Anterior Cruciate Ligament Reconstruction

**Advantages**	**Disadvantages**
Reduction of intra‐articular trauma by mitigating the “explosive” entry effect that could occur with higher diameter drill bits	Sequential drilling takes longer than single drilling
Enables precise correction of the position of the tibial tunnel	Enlarges the final tunnel area by 5% approximately
In cases that anterior cruciate ligament remnant preservation is desired, it minimizes disruption of the native tibial footprint	Repeated or sequential drilling may increase the risk of soft‐tissue irritation and skin damage around the entry site of the tunnel
Whole tibial guide repositioning in cases of minor corrections can be challenging, as reintroduction of the guide pin tends to follow the previous track. In these situations, this technique becomes particularly useful, as it allows for precise correction	The elliptical aperture at the tibial surface could theoretically be enlarged; however, it is comparable in size to that produced by a slightly more horizontal tibial tunnel

Tibial tunnel misplacement remains a critical cause of failure in ACL reconstruction, predisposing to graft impingement and failure.^2^, ^3^ We strongly believe that correct anatomical tunnel placement is paramount to avoid complications and achieve good outcomes. This technique enables us to precisely correct the tunnel position without the need of relocating the whole guide in cases in which small correction is desired. Minor repositioning of the guide pin is often challenging, as when the tibial guide is readjusted and the guide pin is reintroduced, it tends to follow the previous track. It is in these situations that this technique becomes particularly useful, as it allows for enhanced correction. The central tibial footprint position is located 39% from anterior to posterior and 48% mediolaterally in the tibia according to imaging studies.^7^ We follow the anatomic description by Verma et al. for the tibial tunnel (2 ± 0.49 mm anterior to the posterior border of the anterior horn of the lateral meniscus).^6^ This is not the only description reported in the literature. Several anatomical studies have described consistent landmarks to define the center of the ACL tibial footprint: approximately 9 mm posterior to the intermeniscal ligament, and about 15 mm anterior to the posterior cruciate ligament. In relation to the tibial spines, the center has been localized at roughly two‐fifths of the interspinous distance from medial to lateral, and approximately 5 mm anterior to the medial tibial eminence. Lastly, according to Jakob and Amis, the tibial footprint of the ACL is located approximately at 40% from anterior to posterior, ranging from 27% to 60%.^8^, ^9^, ^10^

In summary, we describe a tibial tunnel drilling technique that enables millimetric correction through a 6‐mm preliminary tunnel, facilitating precise anatomic placement, reducing intra‐articular trauma, and preserving ACL remnant fibers when desired. The method allows relocation of the definitive tunnel without repeated guide repositioning, with a negligible 5% increase in tunnel area when upsized to 10 mm. This approach may improve anatomical accuracy and reduce complications associated with tibial tunnel misplacement.

## DISCLOSURES

The authors (R.C.R., D.F.P.) declare the following financial interests/personal relationships which may be considered as potential competing interests: R.C.R. reports a relationship with CONMED Corporation, Stryker, and Smith & Nephew that includes consulting or advisory. D.F.P. reports a relationship with CONMED Corporation, Stryker, and Smith & Nephew Inc that includes consulting or advisory and reports a relationship with International Society of Arthroscopy Knee Surgery and Orthopaedic Sports Medicine and Revista Chilena de Ortopedia y Traumatología that includes board membership. The other authors (F.M.K., R.P.S., C.C.V., M.C.G., R.C.M.) declare that they have no known competing financial interests or personal relationships that could have appeared to influence the work reported in this article.
